# Altered dynamics of the prefrontal networks are associated with the risk for postpartum psychosis: a functional magnetic resonance imaging study

**DOI:** 10.1038/s41398-021-01351-5

**Published:** 2021-05-12

**Authors:** Fabio Sambataro, Giulia Cattarinussi, Andrew Lawrence, Alessandra Biaggi, Montserrat Fusté, Katie Hazelgrove, Mitul A. Mehta, Susan Pawlby, Susan Conroy, Gertrude Seneviratne, Michael C. Craig, Carmine M. Pariante, Maddalena Miele, Paola Dazzan

**Affiliations:** 1 Department of Neuroscience (DNS), Padua Neuroscience Center, University of Padova, Padua, Italy; 2 Department of Psychological Medicine, Institute of Psychiatry, Psychology & Neuroscience, King’s College London, De Crespigny Park, London, UK; 3 National Institute for Health Research (NIHR) Mental Health Biomedical Research Centre at South London and Maudsley NHS Foundation Trust and King’s College London, London, UK; 4 Centre for Neuroimaging Sciences, Institute of Psychiatry, Psychology & Neuroscience, King’s College London, London, UK; 5 South London and Maudsley NHS Foundation Trust Channi Kumar Mother and Baby Unit, Bethlem Royal Hospital, London, UK; 6 National Female Hormone Clinic, Maudsley Hospital, SLaM NHS Foundation Trust, and Institute of Psychiatry, Psychology and Neuroscience, King’s College London, London, De Crespigny Park, London, UK; 7 Perinatal Mental Health Service, St Mary’s Hospital, Imperial College London and Central North West London NHS Foundation Trust, London, UK

**Keywords:** Diagnostic markers, Bipolar disorder

## Abstract

Postpartum psychosis (PP) is a severe mental disorder that affects women in the first few weeks after delivery. To date there are no biomarkers that distinguish which women at risk (AR) develop a significant psychiatric relapse postpartum. While altered brain connectivity may contribute to the risk for psychoses unrelated to the puerperium, this remains unexplored in PP. We followed up 32 AR and 27 healthy (HC) women from pregnancy to 8-week postpartum. At this point, we classified women as AR-unwell (*n* = 15) if they had developed a psychiatric relapse meeting DSM-IV diagnostic criteria, or impacting on daily functioning and requiring treatment, or AR-well (*n* = 17) if they remained asymptomatic. Women also underwent an fMRI scan at rest and during an emotional-processing task, to study within- and between-networks functional connectivity. Women AR, and specifically those in the AR-well group, showed increased resting connectivity within an executive network compared to HC. During the execution of the emotional task, women AR also showed decreased connectivity in the executive network, and altered emotional load-dependent connectivity between executive, salience, and default-mode networks. AR-unwell women particularly showed increased salience network-dependent modulation of the default-mode and executive network relative to AR-well, who showed greater executive network-dependent modulation of the salience network. Our finding that the executive network and its interplay with other brain networks implicated in goal-directed behavior are intrinsically altered suggest that they could be considered neural phenotypes for postpartum psychosis and help advance our understanding of the pathophysiology of this disorder.

## Introduction

Postpartum psychosis (PP) is a severe mental disorder that typically develops within the first 4 weeks after childbirth^1^. Although the absolute prevalence is relatively low, with 0.25–0.6 cases/1000 births, the risk of developing PP is as high as 30–40% in women with a history of bipolar disorder, schizoaffective disorder, and in those with a history PP^2,3^. Still, to date there are no biomarkers that characterise which women at risk are more likely to develop an episode of PP after giving birth.

Although brain magnetic resonance imaging (MRI) markers have been extensively investigated in patients with affective and psychotic disorders unrelated to the puerperium, there are only a case report study that has explored brain function in women with PP^4^, and two MRI studies by our group, in a smaller sample separate from the present study, that investigated the cortical and task-related functional correlates of liability for PP^5,6^. Interestingly, in that sample we found that among women at risk, those who developed an episode of PP had neuromorphological correlates similar to those of patients with psychoses not related to the puerperium, including smaller volumes and cortical surface area of the anterior cingulate, and of parahippocampal, hippocampal, and temporal gyri^5^. However, a functional-MRI working memory task showed a picture different from that seen in psychoses not related to the puerperium, with an increase (rather than a decrease) in the connectivity of the right dorsolateral prefrontal cortex (DLPFC) in women at risk of PP, and specific increases in connectivity between the right DLPFC and ipsilateral middle temporal gyrus in those women who developed an episode of PP^6^.

To obtain crucial information on the intrinsic dysfunction of brain networks, and on how their modulation during specific behavioural processes may be impaired in women at risk of PP, it is essential to study both task-dependent and resting functional connectivity simultaneously. This can help to establish whether the aberrant interplay of brain networks thought to play a central role in other psychiatric disorders is also relevant to vulnerability for PP. Resting-state functional MRI (rs-fMRI) can specifically evaluate the synchrony of blood-oxygen-level-dependent signals of spatially remote brain areas, which is considered to reflect the functional connectivity (FC) of intrinsic brain networks. Using this approach, three large-scale brain networks have been consistently identified: the executive, salience, and default mode (DMN) networks. The executive network has been associated with high-level cognitive functions, including attention control, maintenance, and manipulation of information in working memory and executive task performance^7^. The salience network is important in detecting and integrating salient stimuli, and plays a role in awareness and task-set maintenance^8^, while the DMN is thought to mediate emotional processing, memory, attention, and the introspective mental activities in which humans spontaneously and deliberately engage^9^. Also, a ‘triple network’ model has been proposed, whereby these networks would interact in controlling higher cognitive and affective functions^10^, with the executive network being more active during cognitive and emotional tasks, the DMN showing the opposite activity pattern and the salience network mediating the interplay between the executive network and the DMN.

Altered connectivity within and between these networks has been reported in patients with schizophrenia, bipolar and depressive disorders with psychotic symptoms^11–14^, and these alterations have been associated with cognitive deficits in these disorders^15^. Furthermore, in individuals at risk for psychosis altered connectivity of the DMN, executive and salience networks has also been described^16^, and hyperconnectivity within the executive network has been associated with the attention and working memory impairments often seen in these individuals^17^. As functional connectivity may change dynamically to subserve specific brain processes, it represents a particularly helpful approach in studies of cognitive task performance. In patients with affective disorders, this approach has revealed alterations in networks involved in facial emotional processing, crucial to social functions, during both explicit and implicit emotion tasks^18^. Interestingly, abnormalities in functional connectivity in these patients have also been associated with impairments in working memory, verbal and visual memory, attention, concentration, and processing speed^19^.

Here, we conducted the first investigation of combined resting functional network connectivity and its emotional task-dependent modulation in women at risk of developing PP and in healthy women in the same postpartum period. We used Independent Component Analysis (ICA) to study functional connectivity, as this can decompose fMRI data and extract spatially independent and temporally synchronous activity patterns in the brain, that are functionally connected^20^. ICA is a powerful data-driven approach, capable of estimating large-scale networks without a priori selection of seeds for functional connectivity, which would be required with univariate models. Thus, this technique is best suited to study at risk states, as it does not rely on the haemodynamic response, which could be altered in subjects at risk, in the estimation of overall brain responses. We hypothesised that women at risk of PP would show: (a) increased functional connectivity in the executive, salience, and DMN networks, particularly in association with poorer cognitive performance; and (b) an altered interplay between these large-scale networks involved in emotional regulation and cognitive control. In a set of exploratory analyses, we also tested potential differences in connectivity in those women who did and did not experience a significant psychiatric relapse in the postpartum period.

## Material and methods

### Sample

**Fig. 1:**
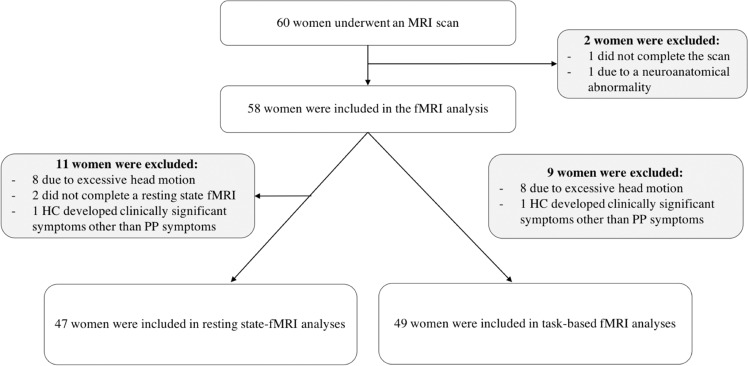
Flow chart of the study. Total number of included and excluded subjects and reasons for exclusion for each fMRI analysis. From the initial sample of 60 women, 47 were included in resting state fMRI and 49 in emotional task fMRI analyses. Sample included in the MRI analyses.

We recruited 32 pregnant women at risk of PP from perinatal mental health services across London. Women were considered to be at risk of PP if 1) they had a diagnosis of bipolar disorder or schizoaffective disorder and/or 2) if they suffered a previous episode of PP (see below). We also recruited 27 control women from antenatal services and advertisements placed in local general practice surgeries, matched to women at risk for age, parity, and handedness. An MRI scan was acquired as close as possible to the 8th week of the postpartum period. All women were recruited in the late second or third trimester of a singleton pregnancy, were at least 18 years of age, and able to communicate in English. Women were excluded if they had any uterine anomaly, known pregnancy complications, severe or relevant chronic medical conditions, or could not undergo an MRI scan. Healthy controls were also negative for any personal history of mental health problems, and a family history of PP. One healthy control was formally diagnosed with Atypical Anorexia Nervosa one year after delivery, although she reported having been underweight before the pregnancy. All participants gave written consent and the study was approved by the South London Research Ethics Committee (10/H0807/14). Sociodemographic and clinical data of the sample are shown in Table 1. Of the 60 women enroled in the study, 47 were included in the resting-state fMRI analyses and 49 in the task-based analyses (Fig. 1).

**Table 1:** Socio-demographic and clinical characteristics and comparisons of the samples.

	AR, *n* = 32	HC, *n* = 27	Statistics AR vs HC	AR-unwell, *n* = 15	AR-well, *n* = 17	Statistics AR-unwell vs AR-well vs HC
Age, mean years (SD)	33.1 (5.2)	33.7 (3.9)	*p* = 0.62 (*t* = 0.51)	32.1 (5.2)	33.9 (5.3)	*p* = 0.51 (*F* = 0.68)
FSIQ, mean (SD)	96.6 (14.9)	105.0 (13.9)	***p*** = **0.03** (*t* = 2.22)	92.1* (16.6)	100.5 (12.3)	***p*** = **0.03** (*F* = 3.94)
Ethnicity (% Caucasian)	62.5 (20)	85.2 (23)	*p* = 0.14 *(χ*^2^ = 3.92)	53.3 (8)	70.6 (12)	*p* = 0.13 *(χ*^2^ = 7.10)
Time between delivery and MRI, mean weeks (SD)	16.4 (6.1)	16.0 (4.8)	*p* = 0.78 (*t* = −0.29)	17.1 (7.4)	15.8 (4.8)	*p* = 0.77 (*F* = 0.27)
Parity (% primiparity)	62.5 (20)	48.1 (13)	*p* = 0.32 *(χ*^2^ = 1.01)	53.3 (8)	70.6 (12)	*p* = 0.34 *(χ*^2^ = 2.19)
Handedness (% right)	93.8 (30)	85.2 (23)	*p* = 0.19 *(χ*^2^ = 3.33)	93.3 (14)	94.1 (16)	*p* = 0.22 *(χ*^2^ = 5.74)
Age of onset, mean years (SD)	20.2 (5.5)	N/A	N/A	20.3 (5.7)	20.1 (5.6)	*p* = 0.92 (*t* = −0.11)
Time between illness onset and MRI, mean years (SD)	13.5 (5.8)	N/A	N/A	12.1 (5.9)	14.7 (5.5)	*p* = 0.22 (*t* = 1.24)
Medications (% yes)^a^	62.5 (20)	N/A	N/A	66.7 (10)	58.8 (10)	*p* = 0.65 *(χ*^2^ = 0.21)
Antipsychotic daily dose, mean CPZ mg equivalents (SD)^a^	260.7 (271.9)	N/A	N/A	287.5 (221.1)	224.9 (348.1)	*p* = 0.69 (*t* = −0.41)
PANSS Total score, mean (SD)^a^	33.4 (4.4)	30.7 (1.1)	***p*** = **0.002** (U = 198.5)	33.9* (5.1)	33.0 (3.9)	***p*** = **0.005** (*K* = 10.67)
YMRS Total score, mean (SD)^a^	0.8 (1.6)	0.3 (0.7)	*p* = 0.14 (U = 324.5)	1.3 (2.2)	0.4 (0.8)	*p* = 0.20 (*K* = 3.23)
HDRS Total score, mean (SD)^a^	5.7 (5.3)	2.7 (1.9)	***p*** = **0.01** (U = 245.0)	6.8* (6.4)	4.8 (4.5)	***p*** = **0.02** (*K* = 7.48)
GAF Total score, mean (SD)^a^	73.6 (12.3)	90.6 (6.2)	***p*** = **0.00** (U = 53.0)	65.9* (3.6)	80.0 (8.9)	***p*** = **0.00** (*K* = 36.9)
CGI Total score, mean (SD)^a^	1.9 (1.3)	1.1 (0.4)	***p*** = **0.00** (U = 227.0)	2.6* (1.5)	1.4 (0.8)	***p*** = **0.00** (*K* = 26.9)

*AR* at risk, *HC* healthy controls, *AR-unwell* at risk women who developed a postpartum relapse, *AR-well* at risk who did not develop a postpartum relapse, *SD* standard deviation, *FSIQ* full scale intelligence quotient, *CPZ* chlorpromazine

*Significant post-hoc comparison relative to HC

^a^At 8-week assessment

Bold values represent statistically significant values (*p* < 0.05)

### Clinical assessment

Current and lifetime diagnoses were obtained at baseline and 8 weeks after delivery. Details of the clinical assessments are provided in the Supplementary Material. The AR women were additionally classified as having a psychiatric relapse (AR-unwell) if, in the first 4 weeks postpartum, they either (a) met DSM-IV diagnostic criteria for Major Depressive Disorder, Bipolar Disorder, Schizoaffective Disorder or Psychosis NOS; or (b) had a combination of DSM-IV symptoms that, whilst not meeting diagnostic criteria, impacted on their daily functioning (e.g. their ability to care for the baby or themselves) and were of sufficient intensity to require a change in treatment (either pharmacological or management plan) (Wesseloo et al.^3^). This broader definition was considered appropriate because all of the AR women were closely monitored by psychiatric services and the majority of them took psychiatric medication to prevent the onset of PP or to treat the symptoms as soon as they developed to prevent them from worsening. Specifically, at the 8-week assessment, 13 women were taking antipsychotics, 4 women lithium, 2 women antidepressants and 1 woman a combination of antidepressants and antipsychotics. We found that 15 of the women at risk developed a psychiatric relapse (AR-unwell) and 17 remained well (AR-well). In the AR-unwell group, eight women had a history of bipolar disorder, three of schizoaffective disorder, one of cyclothymic disorder, and three of previous PP; all women in the AR-well group had a history of bipolar disorder. There were no differences between the AR-unwell and AR-well women in the antipsychotic dose (all p values >0.2).

### Neuropsychological assessment

We used a neuropsychological battery that includes the Wechsler Adult Intelligence Scale-Revised (WAIS-R), Wechsler Test of Adult Reading (WTAR), and Wechsler Memory Scale-III (WMS-III) at the 30th week of gestation. Details of the cognitive domains evaluated, the Z-scores created and the performances in the groups are provided in the Supplementary Material.

### Functional-MRI imaging

#### Imaging acquisition and processing

Details of the data acquisition protocol and image pre-processing can be found in the Supplementary Material. From the total sample, we excluded one woman who did not complete the fMRI acquisition, one due to the presence of a neuroanatomical abnormality, and four that presented excessive head motion. For the resting-state fMRI analyses, we excluded six more women as two did not complete this part of the scan and four presented excessive head motion, leaving 48 scans for inclusion. For the task-based fMRI analyses, we excluded four women because of excessive head motion, leaving 50 scans for inclusion.

### Independent component analysis (ICA)

Two separate spatial group independent component analyses were carried out on resting-state data and task data using GIFT (http://icatb.sourceforge.net) to extract independent components, consisting of spatial maps (SMs) and time courses (TCs) and to evaluate functional network connectivity (FNC). Details of the ICA can be found in the Supplementary Material. This resulted in a final number of 46 intrinsic networks for resting-state analyses and 40 for task analyses, respectively (Table S1, S2) that included the following features for each subject: one spatial map and one power spectrum, calculated from the time course of each intrinsic network, and one between-component functional network connectivity cross-correlation matrix. For each subject in the resting state and task analyses, 46 and 40 spatial maps, 46 and 40 spectra, and a single 46 × 46 and 40 × 40 matrix of FNC were estimated, respectively. Each feature type was concatenated across subjects, thus resulting in separate response matrices per feature type across the whole sample. For resting-state connectivity analyses, we used a MANCOVA approach followed by post-hoc univariate analyses to determine differences between groups in SMs, spectra, and FNC. For task-dependent connectivity, the beta values of linear regressions of the psychophysiological interactions between pairwise TCs and fear presentations were compared using two samples t-test and ANOVAs followed by pairwise post-hoc analyses. Furthermore, connectivity indexes were correlated with cognitive and emotional performance. Connectivity and task-dependent connectivity statistical methods are provided in greater detail in the Supplementary Material.

### Statistical analysis

For imaging, clinical and neuropsychological measures, as data were normally distributed, we calculated mean and standard deviation and estimated between-group differences for continuous variables using one-way ANOVA or t-test and Chi-square as appropriate after varying homoscedasticity. Significance was set at a two-tailed p-value <0.05 corrected for multiple comparisons. We carried out post-hoc analyses when the three-group comparisons were statistically significant. We evaluated within group correlations using Pearson and Spearman (verbal learning and memory domain) coefficients, for parametric and non-parametric data, respectively. Statistical analyses were performed with the Statistical Package for the Social Sciences. Resting-state analyses were carried out using GIFT toolbox.

## Results

### Neuropsychological findings

As we were specifically interested in the interplay between cognitive function and resting state connectivity, we only included in these analyses those women with a usable resting state scan (*n* = 47). We found no differences in full scale IQ between groups (HC, AR: *t* = 1.64 *p* = 0.11; HC, AR-well, AR-unwell: *F* = 1.57, *p* = 0.22). Women AR performed worse than HC in the verbal learning and memory domain (*t* = 2.11, *p* = 0.042) and at trend level in perceptual organisation. There were no statistically significant differences between the AR-unwell and AR-well groups in any neurocognitive domain, although the AR-unwell group performed worse for verbal learning and memory and for attention, concentration and working memory, at trend level significance (see Table S3).

### Resting-state fMRI

**Fig. 2:**
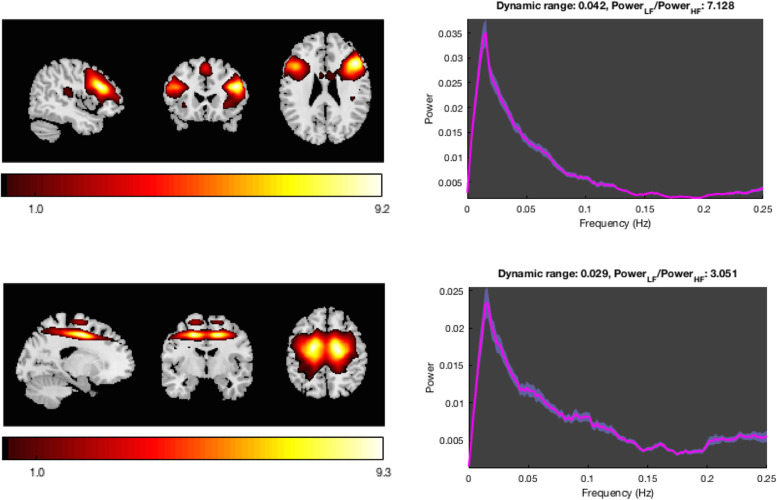
The Intrinsic Networks 53 (top) and 56 (bottom) encompassing executive networks showed a significant effect of diagnosis. Spatial maps of the independent component loadings are overlaid on the Montreal Neurological Institute brain template. Average power spectrum for each frequency (Hz) is measured in arbitrary units (a.u.).

**Fig. 3:**
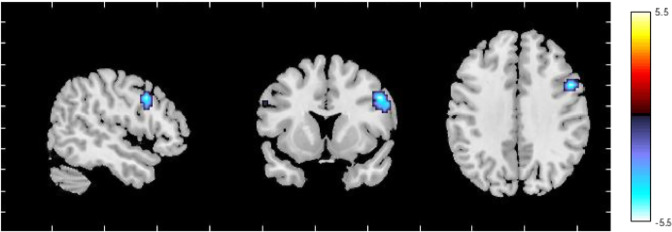
Intrinsic connectivity within the executive network (IN53) was increased in women at risk for postpartum psychosis (AR) relative to control women in the right dorsolateral prefrontal cortex (peak cluster *x*, *y*, *z* = 51,12,36). Probability maps of intrinsic network loadings differences are thresholded at *p* = 0.005 and corrected for multiple comparisons with alpha=0.05 and overlaid on the Montreal Neurological Institute brain template. Colour bar indicates t-scores. IN, Intrinsic Network.

We identified 46 intrinsic networks (Table S1). Multivariate analyses of the spatial maps showed that women at risk of PP had higher connectivity in two intrinsic networks (IN) part of the executive network, compared to women control. The first network (IN53) included the bilateral dorsolateral prefrontal (DLPFC) and parietal cortices, the second (IN56) entailed the bilateral prefrontal cortex (Fig. 2 and Fig. S1). Univariate post-hoc analyses confirmed increased independent component loadings in a cluster in the right DLPFC in AR women within the IN53 network (peak coordinates *x*, *y*, *z* = 51,12,36; Fig. 3). Exploratory analyses confirmed this was present in AR-well women compared to the HC women (p = 0.035) but not in AR-unwell women. Within the IN56 network, no regions survived univariate correction for multiple comparisons. Additionally, we found a significant effect of diagnosis on the spectral of IN75 (salience network), but this result did not survive univariate post-hoc testing. We did not find any significant effect of diagnosis on the spectral and FNC analyses.

Given the role of the executive network in verbal learning and memory, the neurocognitive domains in which AR women performed worse than HC, we run a correlation between the scores in these domains and the right DLPFC cluster average independent component loadings for IN53 and found a positive correlation in AR women (*r* = 0.557, *p* = 0.006). Furthermore, an exploratory analysis additionally showed that in the AR-well, but not in the AR-unwell women, the average independent component loadings of the right DLPFC cluster were significantly positively correlated with verbal learning and memory scores (*ρ* = 0.714; *p* = 0.006) (Fig. S2), indicating that hyperconnectivity of the DLPFC was associated with better cognitive performances in those women at risk who did not have a postpartum episode. Of note, we did not find any correlation between DLPFC and antipsychotic daily dose (*p* > 0.2).

### Facial emotion presentation task

**Fig. 4:**
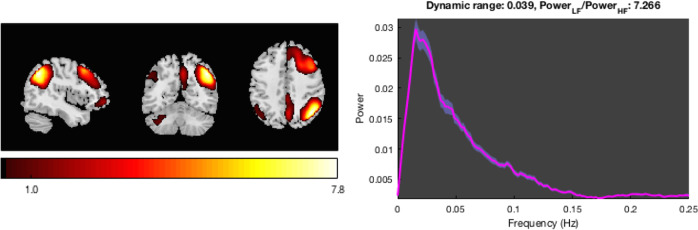
The fear-dependent modulation of the executive networks IC39 time course was reduced in women at risk for postpartum psychosis (AR) relative to control women. Spatial maps of the independent component loadings are overlaid on the Montreal Neurological Institute brain template. Average power spectrum for each frequency (Hz) is measured in arbitrary units (a.u.).

We identified 40 non-artefactual independent components (Tab. S.3). Details of multivariate and univariate analyses are provided in the Supplementary Material. In the analysis of task-dependent connectivity, we found a significant association between the time courses of 14 independent components and the regressors of the emotional task. Given our a priori hypothesis on the role of the triple network model in PP, 11 independent components encompassing executive (*n* = 6), salience (*n* = 3), and DMN (*n* = 2) networks were further compared in women AR and HC to investigate the effect of diagnosis on their network dynamics (Fig. S3). This showed that the synchronisation between the IC39 time course and its fear-dependent modulation was reduced in AR women compared to HC (*p* = 0.020) and that this was present in AR-unwell (*p* = 0.03), but not in AR-well women (Fig. 4).

### Load-dependent inter-network connectivity

**Fig. 5:**
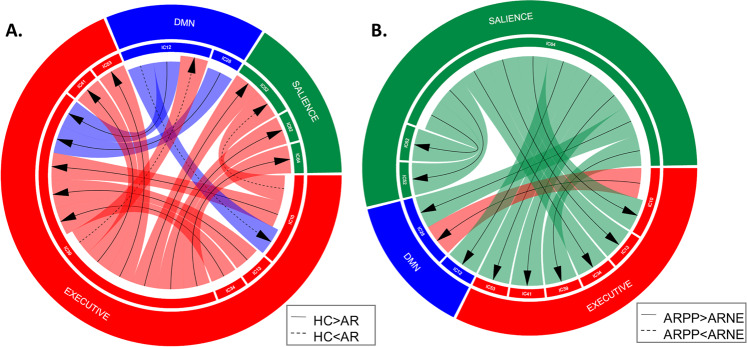
Emotional load-dependent inter-network connectivity differences across diagnosis. **A** Fear load-dependent inter-network connectivity differences between women at risk for postpartum psychosis (AR) and control women (HCs). **B** Fear load-dependent connectivity differences between at risk women who developed (AR-unwell) or did not develop (AR-well) a relapse in the postpartum. Solid lines indicate significant reduced emotional load inter-network connectivity modulation in AR relative to HCs (**A**), and in AR-well relative to AR-unwell (**B**); dotted lines represent increased emotional load inter-network connectivity modulation in AR relative to HCs (**A**), in AR-well relative to AR-unwell (**B**), respectively. Arrows represent the directions of inter-network connectivity. The colours of the bands surrounding the arrows indicate the source of the connectivity. The inner circle indicates the names of the independent components (ICs). The outer circle reports the networks the ICs belong. Executive network is showed in red, salience in green, and default mode network in blue.

We found a significant interaction between fear and time courses of the DMN (IC12, IC28), the salience (IC52, IC62, IC64) and the executive networks (IC10, IC13, IC34, IC41, IC53) in the modulation of the IC39 (executive network) time course (Table S4). In particular, the women AR, compared to the HC, showed reduced fear-dependent modulation from the DMN (IC12, IC28), and the executive networks (IC10, IC13, IC34) on the IC39 time course, as well as from the IC39 to the salience (IC52, IC62, IC64) and other executive subnetworks (IC41, IC53) time courses (Fig. 5A).

Women AR, relative to HC also presented increased fear-dependent modulation from the executive network IC10 to the salience network IC52, and this was found in AR-well women but not in AR-unwell. In a further exploratory analysis in the AR subgroups, we found that AR-unwell women, compared to AR-well women presented an increase of fear-dependent modulation of the IC64 (salience) to the DMN (IC12, IC28), the executive (IC10, IC13, IC34, IC39, IC41, IC53) and other salience networks (IC52, IC62), and of the DMN (IC28) from the executive network (IC10) (Fig. 5B, Table S4).

During the negative emotion facial presentation task, AR women, and both AR subgroups, but not HC women (*p* > 0.2), showed a positive correlation between fear-dependent connectivity and RTs for the IC39 (AR: *r* = 0.423, *p* = 0.040; AR-well: *r* = 0.692, *p* = 0.006; AR-PP: *r* = 0.733, *p* = 0.016). Furthermore, inter-network connectivity between the DMN and the executive (IC12-IC39; IC28-IC39), the executive and the salience (IC39-IC52; IC39-IC62; IC39-IC64) and the executive network and itself (IC10-IC39, IC13-IC39, IC34-IC39, IC39-IC41, IC39-IC53) was significantly inversely correlated with RTs. In particular, the higher the modulation of inter-network connectivity, the lower the RTs (Table S5), suggesting that in AR women a lower inter- and intra-network connectivity was associated with a faster response to emotional stimuli. We did not find any correlation between antipsychotic dose and functional connectivity, load-dependent connectivity, and RTs during task performance, respectively (all *p*’s > 0.2). As we subsequently became aware that one healthy control had developed an eating disorder in the first year of the postpartum, we repeated all analyses for both rs-fMRI and task-based fMRI excluding this participant, but this exclusion did not change our results.

## Discussion

This is the first study to investigate functional connectivity at rest and during emotional processing in women at risk of PP. Our main finding is that women at risk of PP show altered static and dynamic connectivity in brain networks associated with goal-directed behaviour, relative to healthy women. More specifically, we provide the first evidence that an increased intrinsic functional connectivity of the right DLPFC within the executive network, correlated with better pre-partum cognitive performance, may represent a marker of resilience to postpartum relapse, as it is specifically present in those women at risk of PP who remain well. We also find evidence of an impaired emotional regulation in women at risk of PP, as indicated by their reduced emotional-dependent modulation of functional connectivity of the executive network, which correlated with longer reaction time to the presentation of negative emotional stimuli. Interestingly, our data show that it is those women at risk of PP who develop a postpartum relapse who have an increased fear-dependent modulation of the inter-network connectivity from the salience to the DMN networks, the executive and salience subnetworks, as well as from the executive to the salience networks compared to women at risk who remain well, which suggests that alterations in the triple network could represent a state marker for the disease.

The DLPFC is implicated in executive processing, including working memory, attention, decision making, problem-solving, and emotion regulation^21,22^, and has been consistently found to be altered in both structure and function in patients with schizophrenia^23,24^ and bipolar disorder^25^, and in individuals at risk for psychosis^26,27^ and bipolar disorder^27,28^. While alterations of the prefrontal cortex and its associated executive network could reflect a vulnerability to developing psychosis, it is possible that its ability to increase connectivity represents a compensatory neural mechanism that protects from developing the disorder. This is indicated by our finding of an increase in intrinsic DLPFC connectivity (associated with better cognitive performance) in those women at risk who remained well, which is also consistent with similar findings from another study in first degree relatives of patients with schizophrenia who do not develop psychosis^17^.

The executive network was also reduced in synchrony when engaged in fearful facial emotions in women at risk compared to healthy women, and this was significant only in those women who developed a relapse in the first four weeks postpartum. Furthermore, women at risk also showed a reduced temporal relationship between the executive and salience, DMN, and executive subnetworks during fearful emotion presentation, relative to the controls. This could reflect a difficulty in understanding and decoding fear in the women at risk, similar to what is reported in patients with psychoses and bipolar disorder^29,30^. Furthermore, fear-dependent inter-network connectivity between the DMN and the executive, the salience and the executive, and within the executive networks, associated with shorter response times to fear presentation, was reduced in all at risk women. This further suggests that women at risk in general have reduced efficiency of network connectivity and may require a greater ability to modulate inter-network connectivity to perform a simple implicit negative affect regulation task.

Previous neuroimaging studies have reported reduced connectivity within executive, salience, and DMN networks in patients with psychosis and bipolar disorder^14,31^. Our work provides additional evidence of altered connectivity across these brain networks during fearful emotional processing in women at risk for PP, a different clinical population. In this population, these deficits were mostly driven by the women at risk who developed a relapse, with the women at risk who remained well presenting fewer deficits in inter-network connectivity. It is possible that an altered modulation of executive subnetworks, and DMN and salience networks, which are pivotal in the regulation of goal-directed behaviour on the executive network that mediates this behaviour per se, contributes to the risk of, and may protect from developing a psychiatric relapse in the postpartum. We found that within at risk women, only those who remained well had greater connectivity between the salience network (anterior insula, dorsal anterior cingulate cortex) and the executive network (prefronto-parietal cortex) relative to the healthy women. Given the positive association between executive network engagement and more efficient emotional regulation (see below), this suggests that the modulation of salience on the executive network during fearful emotion processing may have a protective role against developing postpartum symptoms in women at risk of PP.

Women at risk who developed a relapse in the postpartum also had increased fear-dependent connectivity from a salience subnetwork to the executive, salience, and DMN networks, and from executive subnetworks to the DMN during fearful emotion processing, compared to women at risk who remained well. This increased fear-dependent connectivity in the women who develop a postpartum relapse may indicate the presence of an aberrant network function in the triple network model described earlier, and which has been implicated in psychosis.

To our knowledge, this is the first study to evaluate cognition in women at risk for PP and to report a worse performance in verbal learning and memory in these women compared to a healthy control group. These findings are consistent with the evidence of similar deficits in both patients with affective and non-affective psychoses unrelated to the puerperium, both in early and late disease stages^32–35^. Interestingly, as mentioned above only in the women at risk who remained well a better cognitive performance was correlated with higher right DLPFC connectivity within the executive network, possibly representing both a protective factor from postpartum relapses and a compensatory mechanism that allows better cognitive performance. Although these findings are intriguing, they should be interpreted with caution as brain function was estimated in the postpartum and the sample size was relatively small.

Differential sensitivity to the rapid post-delivery changes in estrogens concentrations has been proposed as a mechanism to explain the rapid symptom onset in PP^36^. Estrogens affect dopamine signalling and dopamine-dependent cognitive processes, and striatal dopamine D2 receptor binding potential is reduced in women in the postpartum^37,38^. In women at risk for PP, the modulation of dopamine signalling induced by changes in estrogen levels may result in PFC dysfunction, and more generally in executive network alterations^39^. The ability to compensate for this PFC dysfunction with increased connectivity in the executive network (associated with better cognitive performance) could represent a protective mechanism against the onset of psychosis. Dopamine modulates within- and between-network connectivity^40^ and could be involved in the aberrant modulation interplay between the executive and other networks. In women at risk, and particularly in those who develop a postpartum relapse, an altered dopamine signalling could reduce the interaction between the executive, salience, and DMN networks and the executive network, leading to cognitive fragmentation and dysregulation of functional connectivity modulation in response to emotional stimuli. Furthermore, is possible that an increase in dopamine levels in the postpartum in women who develop an episode could boost salience network activity^41^, thus increasing the modulation of this network on the executive, and the DMN networks. It is also possible that the salience network modulates the other brain networks in a non-efficient way, leading to over‐attribution of meaning to irrelevant stimuli and consequent symptoms^42^. Finally, dopamine may counterbalance a hyperactive salience network via increased modulation of the executive network on salience networks, acting as a protective factor against a relapse in women at risk of PP. In summary, in women at risk of PP, an altered response to estrogen levels could lead to a disruption in dopamine signalling which, if not compensated through the modulation of the executive network function, would result in an overactive salience network mediating the onset of symptoms in the postpartum. This different response to dopamine signalling may be due to several factors including genetic and environmental factors and their interplay^43^.

Our study has several strengths and limitations. First, it is important to emphasise that women at PP are simply a very difficult group of subjects to recruit, especially so close to delivery, at times when they tend to be under intense scrutiny from mental health services because of the risk for their health and the health of the baby (at least in the British National Health Services), are struggling with their previous mental illness in the context of the recent arrival of the baby, and, for those who are unwell, they experience the depressive or manic symptoms which are intrinsic to the diagnosis. It is thus not surprising that there are so few biological studies of women in the immediate postpartum, and virtually none using neuroimaging outside our research group. In addition to recruiting such complex participants, we also managed to recruit a population homogeneous for age. However, all of this does not detract from the fact that the sample is relatively small, and the findings need independent replication. The temporal analyses in particular, including the spectral power and FNC, may have benefited from greater statistical power. In terms of methodology, using a comparison group of healthy women in the same postpartum period reduces the chances of identifying functional brain changes related to hormonal or labour-related hemodynamic changes, rather than disease. In view of the lack of previous evidence, ICA is the most appropriate approach to study functional connectivity within- and between-networks, as it does not select a priori seed regions or temporal models. Lastly, studying brain dynamics both at rest and during task performance allows the identification of not only task-related changes in functional connectivity but also of differences that are independent of paradigm conditions. However, not all confounders could be avoided. For example, women in our study were not drug naïve, with 62.5% taking medication at the time of MRI, and drug exposure could be associated with changes in functional connectivity^44^. Reassuringly, we did not find any correlation between antipsychotic dose and functional connectivity and behavioural performance in this study and found no between-group differences in antipsychotic daily dose at the time of MRI. Notably, all four women who were taking lithium remained well in the post-partum, while the two women taking antidepressant medications developed symptoms after delivery. Due to the low number of subjects taking mood stabilisers or antidepressants, we were not able to consider the possible role of these medications in our analyses. An additional limitation is the fact that women at risk who had a relapse had a shorter interval between delivery and MRI acquisition than the other two groups and we cannot exclude this may have had some influence on functional connectivity. Crucially, the MRI could only be acquired in the postpartum, and therefore we cannot comment on functional connectivity before delivery.

This study represents the first step towards achieving a better understanding of functional brain connectivity at rest and during emotional processing as a marker of vulnerability to postpartum episodes. Our findings that the executive network and its interplay with other brain networks implicated in goal-directed behaviour are intrinsically altered suggest that they could be considered neural phenotypes for the disease and help advance our understanding of the pathophysiology of PP.

## Supplementary information

Supplementary material
