# Supplementary material for: Altered dynamics of the prefrontal networks are associated with the risk for postpartum psychosis: a functional magnetic resonance imaging study
Source: Transl Psychiatry. 2021 May 12;11:238. doi: 10.1038/s41398-021-01351-5 (PMC8113224; doi:10.1038/s41398-021-01351-5)
Supplement: Supplementary file 1 — Supplementary material [file 41398_2021_1351_MOESM1_ESM.docx]

Paper submitted to Translational Psychiatry

Revised version

**Supplementary Material**

**Altered dynamics of the prefrontal network are associated with risk for postpartum psychosis: a functional MRI study**

Fabio Sambataro, Giulia Cattarinussi, Andrew Lawrence, Alessandra Biaggi, Montserrat Fusté,

Katie Hazelgrove**,** Mitul A. Mehta, Susan Pawlby, Susan Conroy, Gertrude Seneviratne,

Michael C. Craig, Carmine M. Pariante, Maddalena Miele, Paola Dazzan

**Materials and methods**

**Sample**

All women were recruited in the late second or third trimester of a singleton pregnancy, were at least 18 years of age and able to communicate in English. Women were excluded if they had any uterine anomaly, known pregnancy complications, severe or relevant chronic medical conditions, or could not undergo an MRI scan. Healthy controls were also negative for any personal history of mental health problems, and for a family history of PP. One healthy control developed an eating disorder after the MRI scan.

**Clinical assessment**

Current and lifetime diagnoses were obtained with the Structural Clinical Interview for the Diagnostic and Statistical Manual of Mental Disorders, Fourth Edition (SCID) (DSM-IV) at baseline and 8 weeks after delivery. At MRI, we assessed general functioning with the Global Assessment of Functioning Scale (GAF) and Clinical Global Impression Scale (CGI) while symptoms were assessed with the Positive and Negative Syndrome Scale (PANSS) (1), the Young Mania Rating Scale (YMRS) (2) and the Hamilton Depression Rating Scale (HDRS) (3). The dose of antipsychotic medications at the 8-week follow up was converted into chlorpromazine equivalents (4).

**Neuropsychological assessment**

We evaluated eight neurocognitive domains, using the Wechsler Adult Intelligence Scale – Revised (WAIS-R), Wechsler Test of Adult Reading (WTAR) and Wechsler Memory Scale-III (WMS-III). These included: IQ (WTAR), verbal learning and memory (Logical Memory Immediate and Delayed recall from the WMS-III), visual memory (Visual Reproduction Immediate and Delayed recall of the WMS-III), executive function (Trail Making Test B, Verbal Fluency from the WMS-III), attention, concentration and working memory (Trail Making Test A from the WMS-III and Digit Symbol, Digit Span and Arithmetic from the WAIS-III), speed of processing (Digit symbol from the WAIS-III), verbal comprehension (Similarities and Vocabulary from WAIS-III) and perceptual organization (Block Design from the WAIS-III) (Table S.1). For a detailed description of the scales see Strauss et al. (A Compendium of Neuropsychological Tests: Administration, Norms and Commentary, Third Edition, Oxford University Press, 2006).

With the exception of scores that are already standardized based on population norms (WTAR), we used a regression-based approach to create standards for the neurocognitive measurements. These were calculated by regressing age, gender, ethnicity and level of education on each variable in a group of healthy controls and then creating standard scores from the regression-adjusted scores. Only the Trail Making Test (Parts A and B) presented significant skewness, so the results were log-transformed before standardisation. The same procedure was applied to the patient sample, using the normative standards from the control sample (5).

**Image acquisition**

Scans were acquired at the Centre for Neuroimaging Sciences, King’s College London on a 3T dedicated head scanner (General Electric MR750 3.0T MR) equipped with 40mT/m gradients and a 12-channel head coil. Images were acquired using Gradient Recalled EPI with the following parameters: repeat time (TR) = 2000 ms, echo time (TE) = 30 ms, field of view = 24 cm, acquisition matrix = 64 × 64, flip angle = 75 ̊, voxel size = 3.75 × 3.75 × 3.75 mm3, slice thickness = 3 mm, gap = 3.3 mm, number of slices = 41; descending acquisition order. The resting-state scans lasted 7-minutes during which subjects were asked to lay still with their eyes open while fixing a cross-hair on the screen. The facial emotion presentation task consisted of 60 task trials and 12 fixation trials and lasted for 7-minutes (6). 60 photographs of Caucasian faces representing either varying levels of fear (fear 100% and fear 50%, the latter displaying fear morphed with neutral faces with a 50/50 percent ratio) and neutral expressions were shown. Each poser appeared only once, and stimuli were presented in pseudorandomized order. The subjects were asked to indicate the gender of adult faces (male, female) with a button press and reaction times (RT) were recorded.

**Image pre-processing**

Functional and structural magnetic resonance imaging data pre-processing was performed in Data Processing & Analysis for Brain Imaging (DPABI, http://rfmri.org/dpabi) and Statistical Parametrical Mapping 8 (SPM8) (<http://www.fil.ion.ucl.ac.uk>), running under the MATLAB R2016a (The Mathworks, Sherborn, MA, USA), separately for each scan. First, individual images were visually inspected for anatomical abnormalities and artefacts. Then, images were reoriented into AC-PC space and realigned for head motion correction. Images were then normalized to the Montreal Neurological Institute (MNI) standard brain with a resulting isotropic voxel size of 3x3x3 mm3. Finally, smoothing was carried out with an 8-mm full width half maximum isotropic Gaussian kernel.

**Independent component analysis (ICA)**

Two separate spatial group independent component analyses were carried out on resting state data and task data using the Group ICA of fMRI Toolbox (GIFT3.0b; <http://icatb.sourceforge.net)>. To estimate the independent components we used the Infomax ICA algorithm (7). A two-step principal component analysis was first applied to reduce dimensionality of the data. A high model order ICA with 75 independent components was used in accordance with previous studies that demonstrated that this model yields refined components that correspond to known anatomical and functional segmentations (8,9). Group independent components, consisting of group spatial maps (SMs) and time courses (TCs), were back-reconstructed to individual spatial maps and time courses using GICA3 approach and z-transformed. For each participant, an individual Independent Component spatial map comprises voxel-wise Independent Component loadings that can represent the strength of FC, as they reflect the correspondence between the estimated time course in each voxel for each individual and the time course of the aggregate independent component itself (10). The independent components were screened for reliability as indicated by a coefficient of stability > 0.90 estimated using 50 permutated ICA estimations via ICASSO toolbox (ICASSO; (11). Artefactual patterns identified with a spatial correlation of the spatial maps of R2≤0.005 with gray matter or R2>0.02 for CSF or by visual inspection were excluded. This screening resulted in a final number of 46 intrinsic networks for resting state analyses and 40, for task analyses, respectively (Table S1, S2) that included the following features: one spatial map and time course for each intrinsic network, and one between-component functional network connectivity (FNC) cross-correlation matrix. Thresholded (t>mean+4 SD) spatial maps were used to increase the specificity of each network (12). The multitaper approach as implemented in Chronux in MATLAB (http://www.chronux.org) was used to estimate spectra on detrended time courses (after the mean, slope, and π and 2π period sines and cosines removal). Briefly, detrended, despiked and low-pass filtered (high frequency cut-off = 0.15 Hz) time courses were pairwise correlated using Pearson's correlation. After z-score calculation using Fisher’s transformation they were entered a 46×46 or 40x40 symmetric cross-correlation matrix for each subject for resting state and task fMRI, respectively. For each subject in the resting state analysis, 46 spatial maps, 46 spectra and a single 46×46 matrix of FNC were calculated. For task analysis, we estimated 40 spatial maps, 40 spectra and 40x40 matrix of FNC. Each feature type was concatenated across subjects, thus resulting in separate response matrices per feature type across the whole sample. To reduce the dimensionality and the autocorrelation of the concatenated response matrices for each feature, a principal component analysis with 10 dimensions each was used.

**Connectivity analyses**

First, for each feature type we created a design matrix with the following predictors: age, diagnosis (AR, HC), mean frame-wise displacement and root mean square of motion (13,14). Then, we used the MANCOVAN toolbox within GIFT 3.0 ([http://mialab.mrn.org/software/manvocan/index.html)](http://mialab,mrn.org/software/manvocan/index.html)) to determine differences between groups in spatial maps, spectra and FNC. MANCOVAN uses multivariate analysis of covariance to identify significant predictors within the design matrix. The reduced model includes only the predictors that are significant after correction for multiple comparisons using the false discovery rate (FDR) (15) with α=0.05. Second, we performed univariate analysis within the reduced model to identify specific relationships between diagnosis and spatial map, spectra and FNC for each intrinsic network while partialling out the effects of nuisance covariates. The first eigenvariate of the independent component loadings from significant clusters was extracted and correlated with cognitive domain Z-scores calculated from the neuropsychological battery (see before) to explore brain-behaviour correlations.

**Task-dependent connectivity**

To identify how fear modulated each network and the connectivity between networks, we estimated dynamic changes of FC during the negative emotion facial presentation task, as described elsewhere (16). Briefly, we performed linear regression analyses at the individual subject level between each time course and the facial emotion presentation task conditions (modelled with stick functions convolved with the box-car hemodynamic response as provided in SPM). To evaluate the effect of fear, we calculated ΔFear (Fear 100% - Fear 50%) contrast as a difference of the betas from the linear regression for each subject. We preferred this comparison relative to neutral faces, because previous literature has shown that neutral emotion expressing faces could be misinterpreted as fearful faces (17). To identify the time courses of the intrinsic networks that were associated with fear processing across all subjects, one-sample tests were performed. Only the time courses that were significantly modulated by fear were used in the group analyses for diagnosis. To study the dynamics of between-network functional connectivity during fear, i.e. how a network B modulated a network A depending on fear intensity levels, we conducted linear regression analyses with time course of network A (TC_A_) as response variable, and time course of network B (TC_B_), as task variable (Fear100% vs Fear50% conditions) and the interaction variable (cross product of task and TC_B_) as Z-scored predictors, along the lines of psychophysiological interaction analyses (18*)*. The beta values of these interactions provide an index of fear-dependent connectivity modulation of TC_B_ on TC_A_ and were compared across diagnostic groups using two-sample t-tests (two groups) and ANOVAs (three groups) followed by pairwise post-hoc t-tests, respectively. Furthermore, the beta values of these interactions were correlated with the ΔFear RTs (the difference between reaction times for Fear 100% and Fear 50%, respectively) during the negative emotion facial presentation task to test brain-behaviour associations.

**Facial emotion presentation task**

Multivariate analyses showed that women At Risk had altered static connectivity in four independent components within IC 24 (sensorimotor), IC 17 (visual), IC 38 (executive) and IC 12 (DMN) (Figure S.3) compared to women control. However, these results did not survive univariate post-hoc analyses. We did not find any significant effect of diagnosis on spectral and FNC analyses.

**Table S.1.** Neurocognitive performance in the sample included in the resting-state analyses. All values except full scale Intelligence Quotient (FSIQ) are expressed as z-scores.

|  | **AR**  **n=23** | **HC**  **n=24** | **Statistics**  **AR vs HC** | **AR-unwell n=10** | **AR-well n=13** | **Statistics**  **AR-unwell vs AR-well vs HC** |
| --- | --- | --- | --- | --- | --- | --- |
| **FSIQ** | 100.8 (13.1) | 107.1 (13.0) | p=0.107 (t=1.64) | 98.7 (15.9) | 102.5 (11.0) | p=0.220  (F=1.57) |
| **Cognitive domain:** |  |  |  |  |  |  |
| *Perceptual organization* | -0.29 (1.0) | 0.28 (0.9) | p=0.052  (t=2.02) | -0.25 (0.7) | -0.32 (1.2) | p=0.144  (F=2.02) |
| *Visual memory* | -0.09 (1.1) | 0.09 (0.8) | p=0.523  (t=0.64) | -0.46 (1.1) | 0.20 (1.0) | p=0.199  (F=1.67) |
| *Verbal learning and memory* | -0.29 (1.1) | 0.27 (0.7) | **p=0.042**  (t=2.11) | -0.53 (1.4) | -0.10 (0.8) | p=0.064  (F=2.93) |
| *Speed of processing* | -0.25 (1.0) | 0.24 (0.9) | p=0.096  (t=1.70) | -0.17 (0.9) | -0.3 (1.2) | p=0.239  (F=1.48) |
| *Executive function* | -0.19 (1.0) | 0.27 (0.7) | p=0.100  (U=180.5) | -0.25 (1.2) | -0.14 (0.9) | p=0.238  (K=2.87) |
| *Verbal comprehension* | -0.19 (0.9) | 0.18 (0.9) | p=0.136  (U=206.0) | -0.41 (1.0) | -0.01 (0.8) | p=0.155  (K=3.73) |
| *Attention, concentration and working memory* | -0.11 (0.6) | 0.11 (0.5) | p=0.148  (U=208.0) | -0.40 (07) | 0.1 (0.5) | p=0.053  (K=5.88) |

AR: at risk, HC: healthy controls, AR-well: at risk with no postpartum relapse, AR-unwell: at risk with a postpartum relapse.

**Table S.2.** Intrinsic Networks (INs) estimated during rest fMRI, labelling and description. The description label was estimated using the GIFT labelling tool based on a 14 resting state networks atlas (http://findlab.stanford.edu/functional_ROIs.html) derived from 90 regions of interest (Shirer et al., 2012).

| **IC number** | **Network** | **Description** |
| --- | --- | --- |
| 9 | SAL | Anterior Insula / Dorsal ACC (Anterior Salience Network) |
| 16 | SAL | Anterior Insula / Dorsal ACC (Anterior Salience Network) |
| 36 | SAL | Anterior Insula / Dorsal ACC (Anterior Salience Network) |
| 42 | SAL | Anterior Insula / Dorsal ACC (Anterior Salience Network) |
| 50 | SAL | Anterior Insula / Dorsal ACC (Anterior Salience Network) |
| 60 | SAL | Posterior Insula (Posterior Salience Network) |
| 75 | SAL | Anterior Insula / Dorsal ACC (Anterior Salience Network) |
| 13 | AUD | Auditory Network |
| 24 | BG | Basal Ganglia Network |
| 25 | LAN | Language Network |
| 41 | LAN | Language Network |
| 46 | LAN | Language Network |
| 72 | LAN | Language Network |
| 1 | EXE | DLPFC / Parietal (EXEN Control Network) |
| 14 | EXE | Right DLPFC / Parietal (Right Executive Control Network) |
| 21 | EXE | Left DLPFC / Parietal (Left Executive Control Network) |
| 31 | EXE | Bilateral PFC |
| 32 | EXE | Bilateral PFC |
| 33 | EXE | Left DLPFC / Parietal (Left Executive Control Network) |
| 35 | EXE | Bilateral PFC |
| 37 | EXE | DLPFC / Parietal (Executive Control Network) |
| 43 | EXE | Left DLPFC / Parietal (Left Executive Control Network) |
| 49 | EXE | Bilateral PFC |
| 53 | EXE | DLPFC / Parietal (Executive Control Network) |
| 56 | EXE | Bilateral PFC |
| 62 | EXE | Bilateral PFC |
| 10 | DMN | Retrosplenial Cortex / Medial Temporal Lobe (Ventral Default Mode Network) |
| 18 | DMN | PCC / MPFC (Dorsal Default Mode Network) |
| 19 | DMN | PCC / MPFC (Dorsal Default Mode Network) |
| 20 | DMN | PCC / MPFC (Dorsal Default Mode Network) |
| 23 | DMN | PCC / MPFC (Dorsal Default Mode Network) |
| 27 | DMN | Retrosplenial Cortex / Medial Temporal Lobe (Ventral Default Mode Network) |
| 28 | DMN | PCC / MPFC (Dorsal Default Mode Network) |
| 47 | DMN | Retrosplenial Cortex / Medial Temporal Lobe (Ventral Default Mode Network) |
| 73 | DMN | Retrosplenial Cortex / Medial Temporal Lobe (Ventral Default Mode Network) |
| 4 | VIS | Higher Visual Network |
| 5 | VIS | Primary Visual Network |
| 11 | VIS | Higher Visual Network |
| 26 | VIS | Primary Visual Network |
| 6 | SM | Sensorimotor Network |
| 7 | SM | Sensorimotor Network |
| 8 | SM | Sensorimotor Network |
| 17 | SM | Sensorimotor Network |
| 40 | SM | Sensorimotor Network |
| 45 | SM | Sensorimotor Network |
| 58 | SM | Sensorimotor Network |

ACC: anterior cingulate cortex: AUD: auditory network; BG: basal ganglia network; DLPFC: dorsolateral prefrontal cortex; EXE: executive network; LAN: language network; MPFC: middle prefrontal cortex; PCC: posterior cingulate cortex; PFC: prefrontal cortex; SM: sensorimotor network; SAL: salience network; VIS: visual network; DMN, default mode network.

**Table S.3.** Independent Components (ICs) estimated during emotional fMRI task, labelling and description. The description label was estimated using the GIFT labelling tool that is based on a 14 resting state networks atlas (http://findlab.stanford.edu/functional_ROIs.html) derived from 90 regions of interest (Shirer et al., 2012).

| **IC**  **number** | **Network** | **Description** |
| --- | --- | --- |
| 32 | SAL | Anterior Insula / Dorsal ACC (Anterior Salience Network) |
| 52 | SAL | Anterior Insula / Dorsal ACC (Anterior Salience Network) |
| 62 | SAL | Anterior Insula / Dorsal ACC (Anterior Salience Network) |
| 64 | SAL | Anterior Insula / Dorsal ACC (Anterior Salience Network) |
| 8 | BG | Basal Ganglia Network |
| 27 | LAN | Language Network |
| 37 | LAN | Language Network |
| 51 | LAN | Language Network |
| 1 | EXE | DLPFC / Parietal |
| 10 | EXE | DLPFC / Parietal (Executive Control Network) |
| 13 | EXE | Bilateral prefrontal cortex |
| 19 | EXE | DLPFC / Parietal (Executive Control Network) |
| 23 | EXE | DLPFC / Parietal (Executive Control Network) |
| 29 | EXE | Bilateral prefrontal cortex |
| 34 | EXE | Left DLPFC / Parietal (Left Executive Control Network) |
| 35 | EXE | Left DLPFC |
| 38 | EXE | Bilateral prefrontal cortex |
| 39 | EXE | Right DLPFC / Parietal (Right Executive Control Network) |
| 41 | EXE | Right DLPFC / Parietal (Right Executive Control Network) |
| 44 | EXE | Bilateral prefrontal cortex |
| 53 | EXE | DLPFC / Parietal |
| 57 | EXE | Bilateral prefrontal cortex |
| 6 | DMN | PCC / MPFC (Dorsal Default Mode Network) |
| 12 | DMN | PCC / MPFC (Dorsal Default Mode Network) |
| 14 | DMN | Retrosplenial Cortex / Medial Temporal Lobe (Ventral Default Mode Network) |
| 18 | DMN | Precuneus Network |
| 25 | DMN | PCC / MPFC (Dorsal Default Mode Network) |
| 28 | DMN | PCC / MPFC (Dorsal Default Mode Network) |
| 43 | DMN | PCC / MPFC (Dorsal Default Mode Network) |
| 11 | VIS | Higher VIS Network |
| 17 | VIS | Intraparietal Sulcus / Frontal Eye Fields (Visuospatial Network) |
| 33 | VIS | Primary Visual Network |
| 36 | VIS | Higher Visual Network |
| 47 | VIS | Primary Visual Network |
| 2 | SM | Sensorimotor Network |
| 3 | SM | Sensorimotor Network |
| 5 | SM | Sensorimotor Network |
| 24 | SM | Sensorimotor Network |
| 46 | SM | Sensorimotor Network |
| 54 | SM | Sensorimotor Network |

ACC: anterior cingulate cortex: AUD: auditory network; BG: basal ganglia network; DLPFC: dorsolateral prefrontal cortex; EXE: executive network; LAN: language network; MPFC: middle prefrontal cortex; PCC: posterior cingulate cortex; PFC: prefrontal cortex; SM: sensorimotor network; SAL: salience network; VIS: visual network.

**Table S.4.** Significant differences in emotional load-dependent connectivity across groups.

|  | | | **AR vs HC** | | **AR-well vs HC** | | **AR-unwell vs HC** | |
| --- | --- | --- | --- | --- | --- | --- | --- | --- |
| **IC input** | **IC target** | **Functional Network Connectivity** | **t_48_** | **p** | **t_37_** | **p** | **t_33_** | **p** |
| 12 | 39 | DMN-EXE | -2.773 | 0.007 | -2.262 | 0.029 | -2.311 | 0.027 |
| 28 | 39 | DMN-EXE | -2.485 | 0.016 |  | n.s. | -2.286 | 0.028 |
| 10 | 39 | EXE-EXE | -2.6299 | 0.011 | -2.051 | 0.047 | -2.336 | 0.025 |
| 13 | 39 | EXE-EXE | -2.3708 | 0.021 |  | n.s. | -2.041 | 0.049 |
| 34 | 39 | EXE-EXE | -3.3197 | 0.001 | -2.499 | 0.017 | -2.981 | 0.005 |
| 12 | 10 | DMN-EXE | 2.297 | 0.026 |  | n.s. | 2.089 | 0.044 |
| 12 | 39 | DMN-EXE | 2.282 | 0.026 |  | n.s. |  | n.s. |
| 10 | 52 | EXE-SAL | 2.092 | 0.041 | 2.591 | 0.013 |  | n.s. |
| 39 | 41 | EXE-EXE | -2.467 | 0.017 |  | n.s. |  | n.s. |
| 39 | 53 | EXE-EXE | -2.568 | 0.013 |  | n.s. | -2.4418 | 0.020 |
| 39 | 52 | EXE-SAL | -2.211 | 0.031 |  | n.s. |  | n.s. |
| 39 | 62 | EXE-SAL | -2.549 | 0.014 | -2.027 | 0.049 | -2.1102 | 0.042 |
| 39 | 64 | EXE-SAL | -2.463 | 0.017 |  | n.s. | -2.3821 | 0.023 |

|  | | | **AR-well vs AR-unwell** | |
| --- | --- | --- | --- | --- |
| **IC input** | **IC target** | **Functional Network Connectivity** | **t_24_** | **p** |
| 64 | 39 | SAL-EXE | -2.712 | 0.012 |
| 64 | 28 | SAL-DMN | -2.8763 | 0.008 |
| 64 | 41 | SAL-EXE | -2.5797 | 0.016 |
| 64 | 53 | SAL-EXE | -3.1951 | 0.003 |
| 10 | 28 | EXE-DMN | -2.5385 | 0.018 |
| 64 | 12 | SAL-DMN | -2.5848 | 0.016 |
| 64 | 10 | SAL-EXE | -2.9066 | 0.007 |
| 64 | 13 | SAL-EXE | -2.7698 | 0.010 |
| 64 | 34 | SAL-EXE | -2.6876 | 0.012 |
| 64 | 52 | SAL-SAL | -2.9456 | 0.007 |
| 64 | 62 | SAL-SAL | -2.6718 | 0.013 |

AR: at risk, HC: healthy controls, AR-well: at risk with no postpartum relapse, AR-unwell: at risk with a postpartum relapse, DMN: default-mode network, EXE: executive network, SAL: salience network.

**Table S.5.** Significant (p>0.05) correlations between load-dependent connectivity and ΔFear reaction times (RTs) across groups.

| **IC input** | **IC target** | **Functional Network Connectivity** | **Statistics** | **HC** | **AR** | **AR-well** | **AR-unwell** |
| --- | --- | --- | --- | --- | --- | --- | --- |
| 12 | 39 | DMN-EXE | r |  | -0.444 | -0.644 | -0.673 |
|  |  |  | p | n.s. | 0.030 | 0.013 | 0.033 |
| 28 | 39 | DMN-EXE | r |  | -0.478 | -0.714 | -0.721 |
|  |  |  | p | n.s. | 0.018 | 0.004 | 0.019 |
| 10 | 39 | EXE-EXE | r |  | -0.471 | -0.705 | -0.733 |
|  |  |  | p | n.s. | 0.020 | 0.005 | 0.016 |
| 13 | 39 | EXE-EXE | r |  | -0.496 | -0.684 | -0.685 |
|  |  |  | p | n.s. | 0.014 | 0.007 | 0.029 |
| 34 | 39 | EXE-EXE | r |  | -0.521 | -0.714 | -0.794 |
|  |  |  | p | n.s. | 0.009 | 0.004 | 0.006 |
| 10 | 52 | EXE-SAL | r |  | -0.495 | -0.648 | -0.673 |
|  |  |  | p | n.s. | 0.014 | 0.012 | 0.033 |
| 39 | 41 | EXE-EXE | r |  | -0.495 | -0.648 | -0.673 |
|  |  |  | p | n.s. | 0.014 | 0.012 | 0.033 |
| 39 | 53 | EXE-EXE | r |  | -0.454 | -0.767 | -0.721 |
|  |  |  | p | n.s. | 0.026 | 0.001 | 0.019 |
| 39 | 52 | EXE-SAL | r |  | -0.461 | -0.640 | -0.721 |
|  |  |  | p | n.s. | 0.023 | 0.014 | 0.019 |
| 39 | 62 | EXE-SAL | r |  | -0.432 | -0.692 | -0.685 |
|  |  |  | p | n.s. | 0.035 | 0.006 | 0.029 |
| 39 | 64 | EXE-SAL | r |  | -.397 | -0.578 | -0.661 |
|  |  |  | p | n.s. | 0.055 | 0.030 | 0.038 |

AR: at risk, HC: healthy controls, AR-well: at risk with no postpartum relapse, AR-unwell: at risk with a postpartum relapse, DMN: default-mode network, EXE: executive network, SAL: salience network.

**Figure S.1.** Design matrix showing the effect of predictors (age, diagnosis, mean frame-wise displacement and root mean square of motion) on the MANCOVA on the spatial maps (left) and on spectra (right) during resting state. Intrinsic Networks (INs) are grouped by type of network.

**
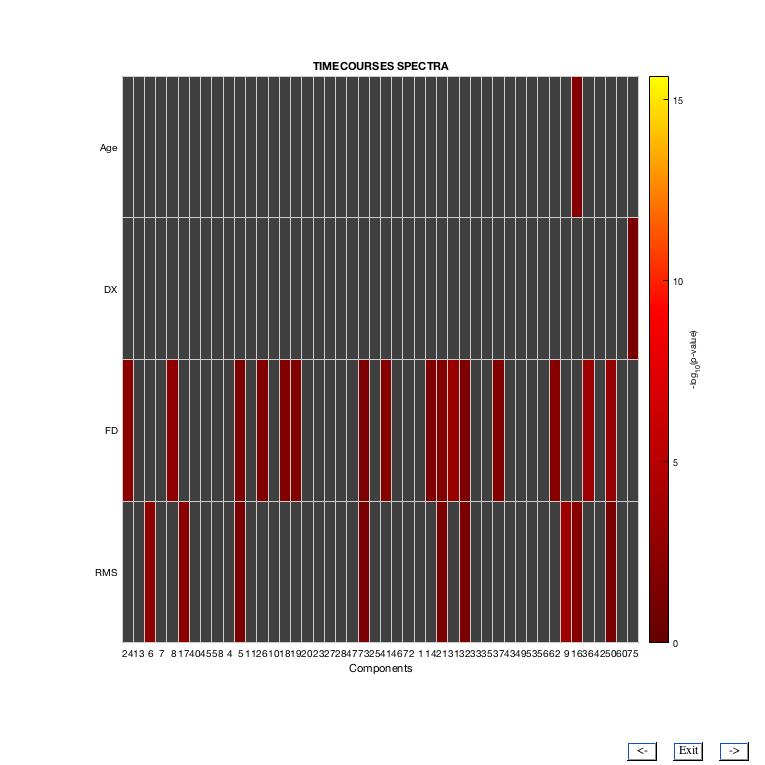
**


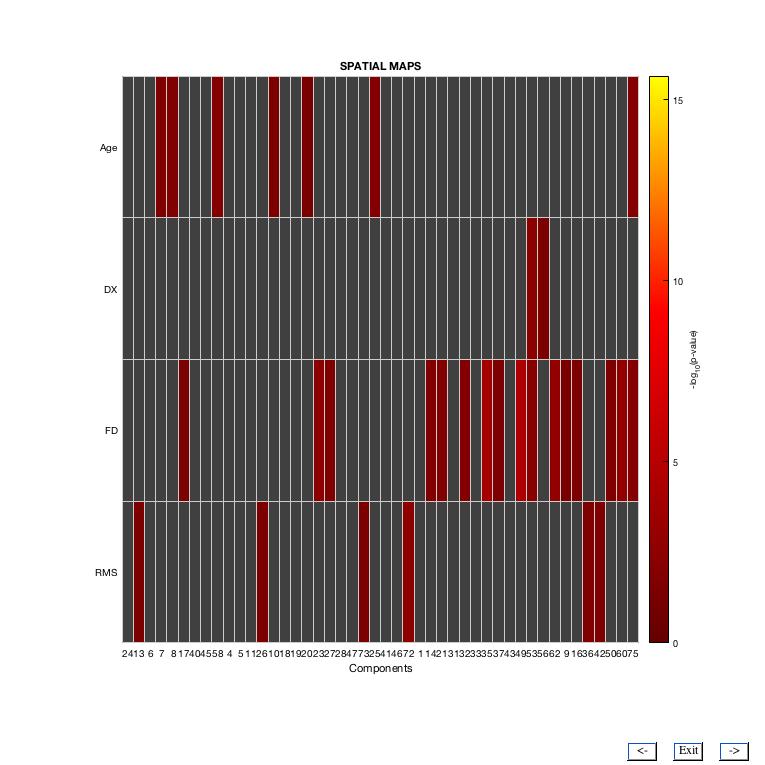
Numbers at the bottom indicate the IN number. Colour bar indicates –log10 (p-value). Dx: diagnosis, FD: mean frame-wise displacement, RMS: root mean square of motion.

**Figure S.2.** Correlations between DLPFC IC loadings from the cluster showing an effect of diagnosis of intrinsic network IN53 and cognitive performance domains. A) correlation between DLPFC signal and verbal learning and memory domain Z-score in AR and HC women; B) correlation between DLPFC signal and verbal learning and memory domain Z-score in AR-well, AR-unwell and HC women.

A B


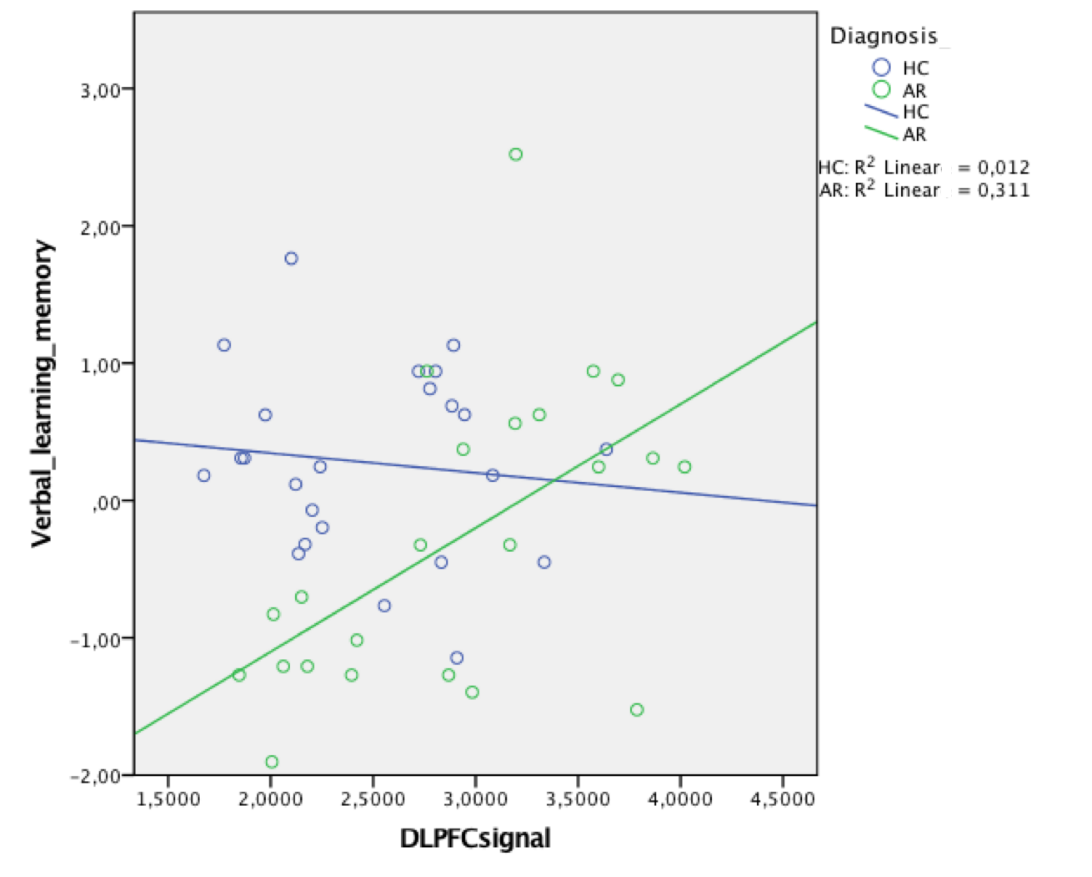

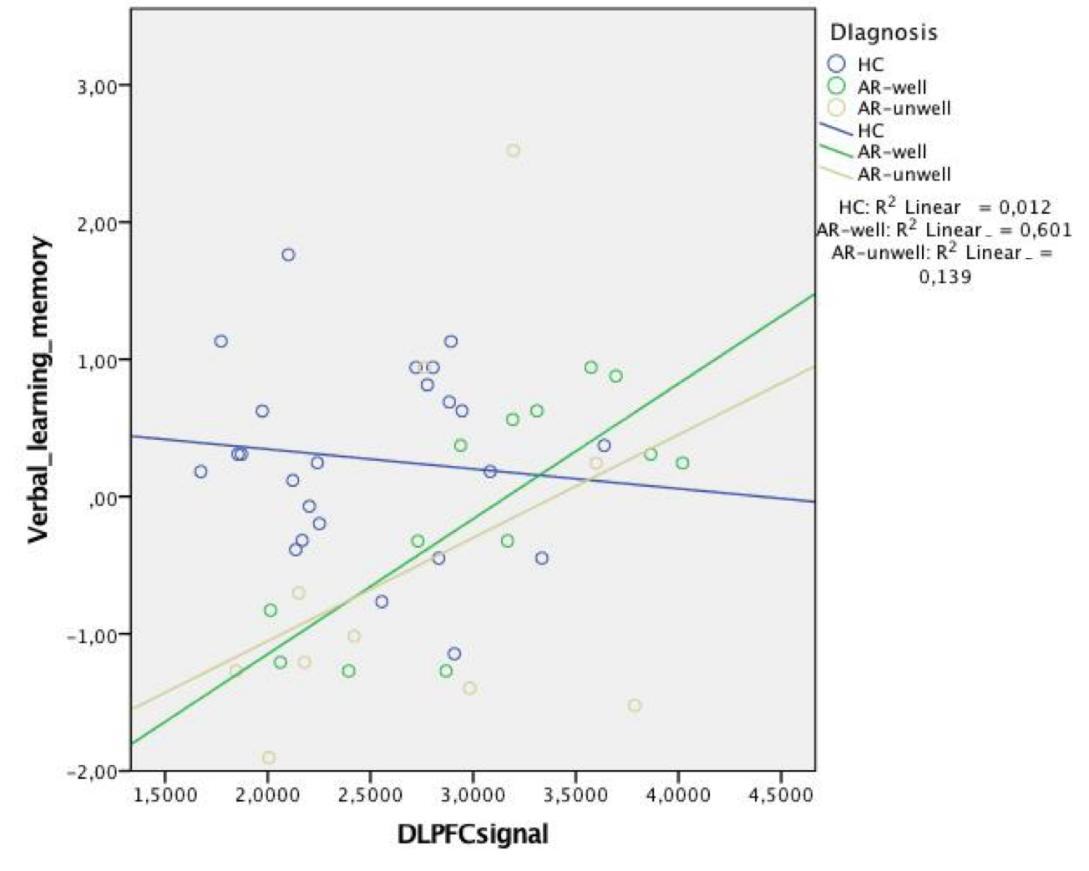


**Figure S.3** Design matrix showing the effect of predictors (age, diagnosis, mean frame-wise displacement and root mean square of motion) on the MANCOVA on the spatial maps (left) and on spectra (right) during the emotional fMRI task. Independent components (ICs) are grouped by type of network. Numbers at the bottom indicate the IC number. Color bar indicates –log10 (p-value). Dx: diagnosis, FD: mean frame-wise displacement, RMS: root mean square of motion.


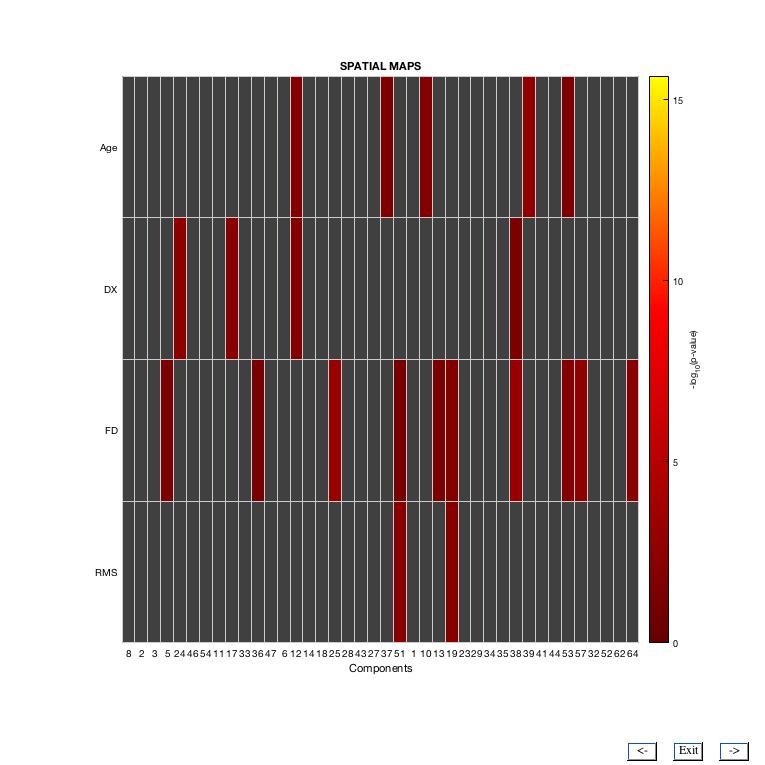

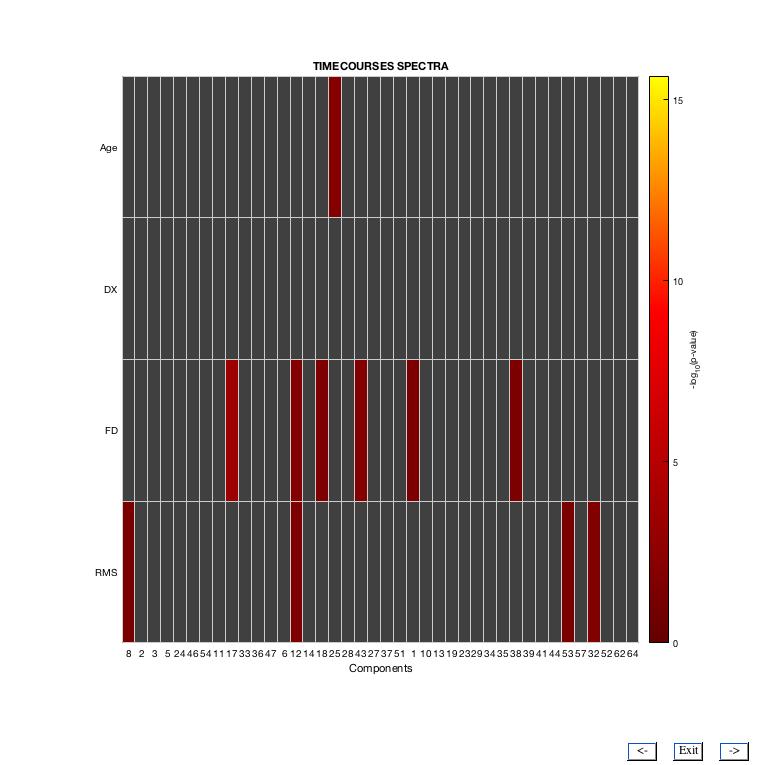


**Figure S.4.** Spatial maps of the Independent Components (ICs) showing a significant effect of diagnosis for fear-dependent connectivity (see Table S.3 for details). Maps display one sample t-tests across all subjects. Color bar indicates t-score.

Default Mode Networks


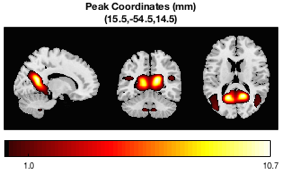
IC 12

Executive Networks


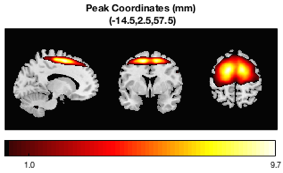
IC 10


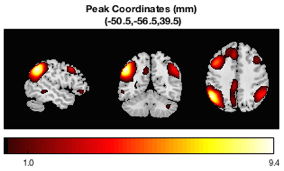
IC 34


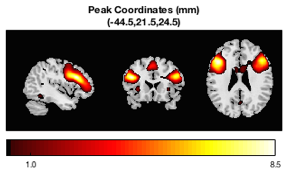
IC 41

Salience Networks


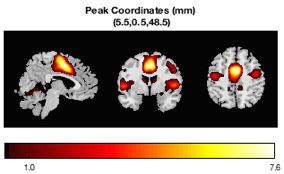
IC52


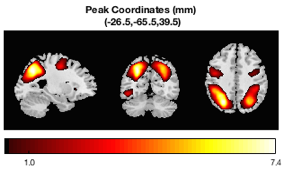
 IC 64


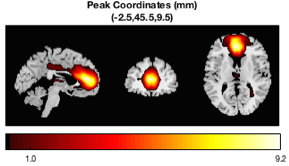
IC 28


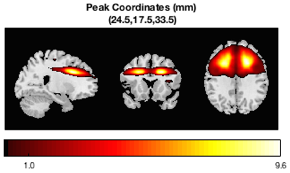
IC 13


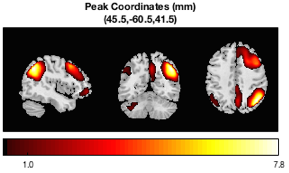
IC 39


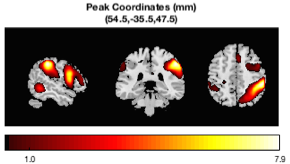
IC 53


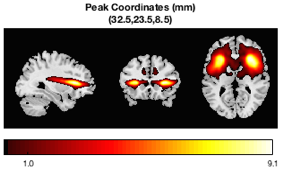
IC 62

**References**

1. Kay, S. R., Fiszbein, A. & Opler, L. A. The positive and negative syndrome scale (PANSS) for schizophrenia. *Schizophr Bull* **13**, 261-276 (1987).
2. Young, R. C., Biggs, J. T., Ziegler, V. E. & Meyer, D. A. A rating scale for mania: reliability, validity and sensitivity. *Br J Psychiatry* **133**, 429-435 (1978).
3. Hamilton, M. A rating scale for depression. *J Neurol Neurosurg Psychiatry* **23**, 56-62. (1960).
4. Woods, S. W. et al.. Best practices: racial and ethnic effects on antipsychotic prescribing practices in a community mental health center. *Psychiatr Serv* **54**, 177-179 (2003).
5. Zanelli, J. et al. Gender differences in neuropsychological performance across psychotic disorders-a multi-centre population based case-control study. *PLoS One* **8**, e77318 (2013).
6. Derntl, B., Kryspin-Exner, I., Fernbach, E., Moser, E. & Habel, U. Emotion recognition accuracy in healthy young females is associated with cycle phase. *Horm Behav* **53**, 90-95 (2008).
7. Bell, A. J. & Sejnowski, T. J.. An information-maximization approach to blind separation and blind deconvolution. *Neural Comput* **7**, 1129-1159 (1995).
8. Kiviniemi, V. et al. Functional segmentation of the brain cortex using high model order group PICA. *Hum Brain Mapp* **30**, 3865-3886 (2009).
9. Smith, S. M. et al. Correspondence of the brain's functional architecture during activation and rest. *Proc Natl Acad Sci U S A* **106**, 13040-13045 (2009).
10. Sambataro, F. et al. Altered dynamics of brain connectivity in major depressive disorder at-rest and during task performance. *Psychiatry Res Neuroimaging* **259**, 1-9 (2017).
11. Himberg, J., Hyvarinen, A. & Esposito, F. Validating the independent components of neuroimaging time series via clustering and visualization. *Neuroimage* **22**, 1214-1222 (2004).
12. Allen, E. A. et al. A baseline for the multivariate comparison of resting-state networks. *Front Syst Neurosci* **5**, 2 (2011).
13. Power, J. D., Barnes, K. A., Snyder, A. Z., Schlaggar, B. L. & Petersen, S. E. Spurious but systematic correlations in functional connectivity MRI networks arise from subject motion. *Neuroimage,* **59**, 2142-2154 (2012).
14. Van Dijk, K. R., Sabuncu, M. R., & Buckner, R. L. The influence of head motion on intrinsic functional connectivity MRI. *Neuroimage* **59**, 431-438 (2012).
15. Genovese, C. R., Lazar, N. A., & Nichols, T. Thresholding of statistical maps in functional neuroimaging using the false discovery rate. *Neuroimage* **15**, 870-878. (2002).
16. Cassidy, C. M. et al. Dynamic Connectivity between Brain Networks Supports Working Memory: Relationships to Dopamine Release and Schizophrenia. *J Neurosci* **36**, 4377-4388 (2016).
17. Hall, J. et al. Overactivation of fear systems to neutral faces in schizophrenia. *Biol Psychiatry* **64**, 70-73 (2008).
18. Friston, K. J. et al. Psychophysiological and modulatory interactions in neuroimaging. *Neuroimage* **6**, 218-229. (1997).
